# Alloying effect on the order–disorder transformation in tetragonal FeNi

**DOI:** 10.1038/s41598-021-84482-5

**Published:** 2021-03-04

**Authors:** Li-Yun Tian, Oliver Gutfleisch, Olle Eriksson, Levente Vitos

**Affiliations:** 1 Applied Materials Physics, Department of Materials Science and Engineering, Royal Institute of Technology, 100 44 Stockholm, Sweden; 2 Division of Materials Theory, Department of Physics and Astronomy, Uppsala University, Box 516, 751 20 Uppsala, Sweden; 3 Institute of Materials Science, Technische Universität Darmstadt, 64287 Darmstadt, Germany; 4 School of Science and Engineering, Örebro University, Örebro, Sweden; 5 Research Institute for Solid State Physics and Optics, Wigner Research Center for Physics, Budapest, 1525 Hungary

**Keywords:** Energy science and technology, Materials science

## Abstract

Tetragonal (${\hbox{L1}}_{0}$) FeNi is a promising material for high-performance rare-earth-free permanent magnets. Pure tetragonal FeNi is very difficult to synthesize due to its low chemical order–disorder transition temperature ($\approx {593}$ K), and thus one must consider alternative non-equilibrium processing routes and alloy design strategies that make the formation of tetragonal FeNi feasible. In this paper, we investigate by density functional theory as implemented in the exact muffin-tin orbitals method whether alloying FeNi with a suitable element can have a positive impact on the phase formation and ordering properties while largely maintaining its attractive intrinsic magnetic properties. We find that small amount of non-magnetic (Al and Ti) or magnetic (Cr and Co) elements increase the order–disorder transition temperature. Adding Mo to the Co-doped system further enhances the ordering temperature while the Curie temperature is decreased only by a few degrees. Our results show that alloying is a viable route to stabilizing the ordered tetragonal phase of FeNi.

## Introduction

The introduction of neodymium magnets in 1984 was a great leap in magnetic materials^1^ and open up many new applications in industry^2^. They are the strongest class of permanent magnets currently available commercially. The downside of this type of magnets, however, is that they include costly rare-earth elements^3–6^, that also have the troubling aspect of being difficult to mine without large environmental imprint. Because of these issues, there is strong research interest to develop highly performant permanent magnets that would not depend on the expensive rare-earth elements^7–9^. One of the promising candidates that has emerged is tetragonal ${\hbox{L1}}_{{0}}$ FeNi (tetrataenite), which is known to have large uniaxial magnetic anisotropy, $K_{u} = 7.0 \times 10^5\,{\hbox{J/m}}^3$^10^, and high Curie temperature (${\hbox{T}}_{\mathrm{c}} \ge {823}$ K)^11^. While these properties are excellent, the problem is that they are unique for the chemically ordered phase of FeNi and currently there is no efficient way to manufacture the ordered phase due to the low chemical order–disorder transition temperature, ${\hbox{T}}_{\mathrm{od}} \approx {593}$ K^12^. This temperature is simply too low to allow fast enough growth of the ordered phase. Different experimental and theoretical solutions to overcome the problem, such as nitrogen insertion^13,14^, are being investigated.

In our previous paper^15^ we showed that density functional theory (DFT) as implemented in the Exact Muffin-Tin Orbitals (EMTO) method^16–18^ gives an accurate theoretical description of tetragonal FeNi alloys. Our theoretical method predicted the chemical order–disorder transition temperature to be 559 K, which differs from the experimentally value by 34 K (6%) only. The method is a theoretical platform that allows to study how the desirable ordered phase of FeNi can be stabilized at elevated temperatures, which would allow the ordered FeNi phase to be grown in a reasonable amount of time. In the present investigation, we consider the effect of alloying on the properties of tetragonal FeNi. It is our expectation that alloying helps to solve the problems with succesfully synthesizing functional FeNi permanent magnets. Our selection of alloying elements include Al, Ti, Cr, Co, and Mo as representative simple metal, non-magnetic and magnetic 3*d* metals and a refractory metal, respectively. We study how alloying affects the lattice constants, magnetic moments, Curie temperature, and order–disorder transition temperature. We find that the order–disorder temperature can be increased by suitable choice of the alloying elements and doping concentrations. We therefore predict that selective alloying is a potential way to make synthesizing high-performance magnetic FeNi phase more achievable.

## Results

**Figure 1:**
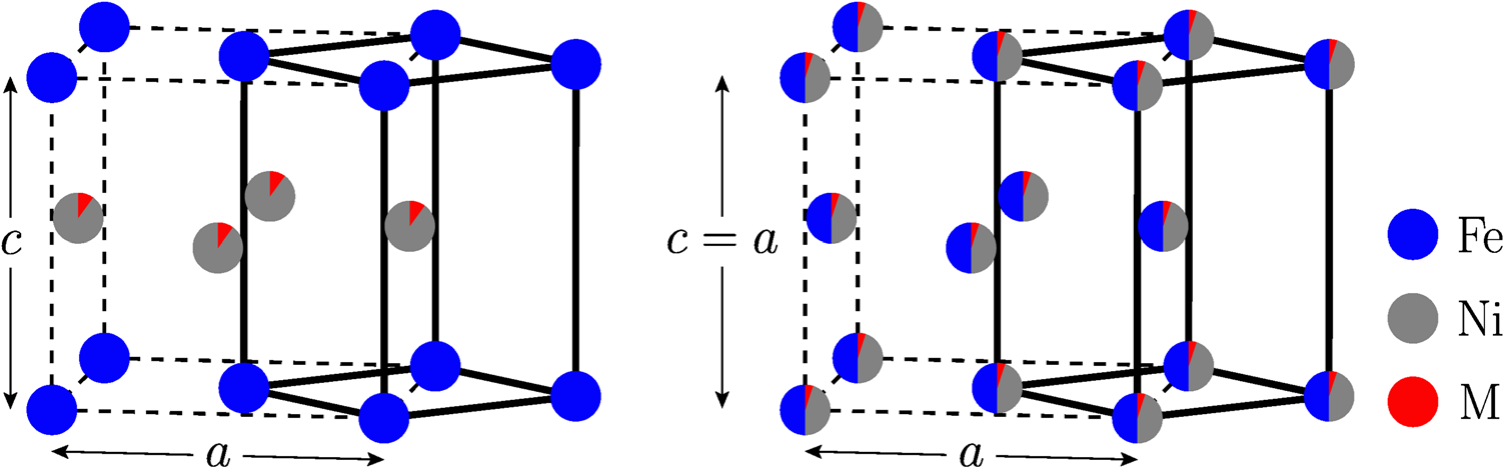
Left panel shows the alloyed ${{\hbox{L1}}}_{0}\,{{\hbox{FeNi}}}_{1-x}M_{x}$ (*M* = Al, Ti, Cr, Co, Mo) structure ($\eta = 1$) and the right panel shows the fully random (fcc) structure ($\eta = 0$). Blue atoms are Fe and gray atoms Ni. The letter *M* and the red part of the atomic sphere indicate the alloying element and its concentration. The convention for the *a* and *c* lattice parameters is shown.

In order to keep the magnetic performance of tetragonal FeNi, only small amounts of *M* substitutions on the Fe/Ni site are adopted. Namely, we consider ${\hbox{Fe}}_{1-x}{M}_{x}{\text{Ni}}$ and ${\hbox{FeNi}}_{1-x}{M}_{x}$ with $x = 0.05$, 0.10 and ${M} = {\hbox{Al}}$, Ti, Cr, Co as well as ${\hbox{Fe}}_{0.98} {\hbox{Mo}}_{0.02}{\text{Ni}}$, ${\hbox{FeNi}}_{0.98} {\hbox{Mo}}_{0.02}$, ${\hbox{Fe}}_{0.93} {\hbox{Co}}_{0.05} {\hbox{Mo}}_{0.02}{\text{Ni}}$ and ${\hbox{Fe}}_{0.95} {\hbox{Co}}_{0.05} {\hbox{Ni}}_{0.98} {\hbox{Mo}}_{0.02}$. The structures of tetragonal *M*-doped FeNi are treated as ${\hbox{Fe}}_{1-x}{M}_{x}{\text{Ni}}$ and ${\hbox{FeNi}}_{1-x}{M}_{x}$ meaning that either the Fe or the Ni layer is doped in the ordered ${\hbox{L}}1_{0}$ phase. The disordered sites are described with the coherent potential approximation (CPA)^19,20^. The tetragonal ${\hbox{L1}}_{0}$ and the fully disordered structures of ${\hbox{FeNi}}_{1-x}{M}_{x}$ are illustrated in Fig. 1. The left panel shows the substitutional *M* doping of the Ni layer in the ${\hbox{L1}}_{0}$ structure, and the right panel shows the chemically disordered face centered cubic (fcc) structure with homogeneous occupation by the three components. The degree of Fe–Ni disorder is measured by the so-called long-range order parameter $\eta$^15^, which has values between 0 and 1, corresponding to the fully random and ordered structures, respectively. Ordering effects on the *M*-doped sublattice in the ${\hbox{L}}1_{0}$ phase are not considered, that is we consider the situation when *M* randomly occupies either the Fe or the Ni sublattice.

**Figure 2:**
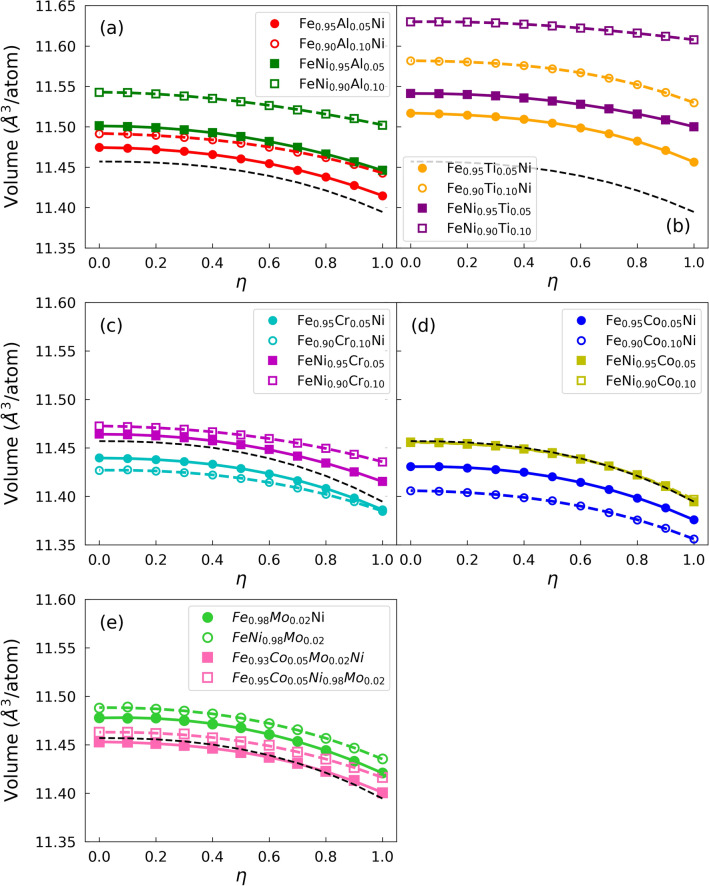
The volume dependence of the long-range order parameter $\eta$ for the ${{\hbox{Fe}}}_{1-x}M_{x}{{\text{Ni}}}$ and ${{\hbox{FeNi}}}_{1-x}{M}_{x}$ ($x = 0.05$, 0.10) (${M} = {{\hbox{Al}}}$, Ti, Cr, Co), and ${{\hbox{Fe}}}_{0.98} {{\hbox{Mo}}}_{0.02}{{\text{Ni}}}$, ${{\hbox{FeNi}}}_{0.98} {{\hbox{Mo}}}_{0.02}$, ${{\hbox{Fe}}}_{0.93} {{\hbox{Co}}}_{0.05} {{\hbox{Mo}}}_{0.02}{{\text{Ni}}}$ and ${{\hbox{Fe}}}_{0.95} {\hbox{Co}}_{0.05} {\hbox{Ni}}_{0.98} {\hbox{Mo}}_{0.02}$ with the EMTO + CPA method. The black dashed line indicates the equilibrium lattice parameter of ${\hbox{L1}}_{0}$ FeNi versus the long-range order parameter $\eta$.

**Table 1:** The lattice parameters (in units of Å) of ${\hbox{Fe}}_{1-x}{M}_{x}{\text{Ni}}$ and ${\hbox{FeNi}}_{1-x}{M}_{x}$ ($x = 0.05$, 0.10) (${M} = {\hbox{Al}}$, Ti, Cr, Co), and ${\hbox{Fe}}_{0.98} {\hbox{Mo}}_{0.02}{\text{Ni}}$, ${\hbox{FeNi}}_{0.98} {\hbox{Mo}}_{0.02}$, ${\hbox{Fe}}_{0.93} {\hbox{Co}}_{0.05} {\hbox{Mo}}_{0.02}{\text{Ni}}$ and ${\hbox{Fe}}_{0.95} {\hbox{Co}}_{0.05} {\hbox{Ni}}_{0.98} {\hbox{Mo}}_{0.02}$ as a function of chemical composition for $L1_{0}$ structures ($\eta$ = 1) and the volume changes relative to undoped ${\hbox{L}}1_{0}$ FeNi expressed as $\Delta$ = $\frac{V - V_{\mathrm {FeNi}}}{V_{\mathrm {FeNi}}} \times 100 \%$.

Structures	*a*	*c*/*a*	$\Delta (\%)$	Structures	*a*	*c*/*a*	$\Delta (\%)$
${\hbox{Fe}}_{0.95} {\hbox{Al}}_{0.05}{\text{Ni}}$	3.570	1.004	0.179	${\hbox{Fe}}_{0.90} {\hbox{Al}}_{0.10}{\text{Ni}}$	3.579	0.998	0.424
${\hbox{Fe}}_{0.95} {\hbox{Ti}}_{0.05}{\text{Ni}}$	3.567	1.010	0.544	${\hbox{Fe}}_{0.90} {\hbox{Ti}}_{0.10}{\text{Ni}}$	3.573	1.011	1.188
${\hbox{Fe}}_{0.95} {\hbox{Cr}}_{0.05}{\text{Ni}}$	3.554	1.014	− 0.071	${\hbox{Fe}}_{0.90} {\hbox{Cr}}_{0.10}{\text{Ni}}$	3.547	1.021	− 0.085
${\hbox{Fe}}_{0.95} {\hbox{Co}}_{0.05}{\text{Ni}}$	3.560	1.008	− 0.162	${\hbox{Fe}}_{0.90} {\hbox{Co}}_{0.10}{\text{Ni}}$	3.556	1.010	− 0.337
${\hbox{Fe}}_{0.98} {\hbox{Mo}}_{0.02}{\text{Ni}}$	3.563	1.010	0.235	${\hbox{Fe}}_{0.93} {\hbox{Co}}_{0.05} {\hbox{Mo}}_{0.02}{\text{Ni}}$	3.558	1.012	0.054
${\hbox{FeNi}}_{0.95} {\hbox{Al}}_{0.05}$	3.557	1.017	0.456	${\hbox{FeNi}}_{0.90} {\hbox{Al}}_{0.10}$	3.553	1.026	0.944
${\hbox{FeNi}}_{0.95} {\hbox{Ti}}_{0.05}$	3.564	1.016	0.929	${\hbox{FeNi}}_{0.90} {\hbox{Ti}}_{0.10}$	3.565	1.025	1.872
${\hbox{FeNi}}_{0.95} {\hbox{Cr}}_{0.05}$	3.566	1.007	0.185	${\hbox{FeNi}}_{0.90} {\hbox{Cr}}_{0.10}$	3.568	1.007	0.362
${\hbox{FeNi}}_{0.95} {\hbox{Co}}_{0.05}$	3.570	1.002	0.0005	${\hbox{FeNi}}_{0.90} {\hbox{Co}}_{0.10}$	3.576	0.997	0.022
${\hbox{FeNi}}_{0.98} {\hbox{Mo}}_{0.02}$	3.566	1.009	0.361	${\hbox{Fe}}_{0.95} {\hbox{Co}}_{0.05} {\hbox{Ni}}_{0.98} {\hbox{Mo}}_{0.02}$	3.563	1.010	0.193
FeNi	3.564	1.007					

The volumes per atom of ${\hbox{Fe}}_{1-x}{M}_{x}{\text{Ni}}$ and ${\hbox{FeNi}}_{1-x}{M}_{x}$ ($x = 0.05$, 0.10) (${M} = {\hbox{Al}}$, Ti, Cr, Co), as well as ${\hbox{Fe}}_{0.98} {\hbox{Mo}}_{0.02}{\text{Ni}}$, ${\hbox{FeNi}}_{0.98} {\hbox{Mo}}_{0.02}$, ${\hbox{Fe}}_{0.93} {\hbox{Co}}_{0.05} {\hbox{Mo}}_{0.02}{\text{Ni}}$ and ${\hbox{Fe}}_{0.95} {\hbox{Co}}_{0.05} {\hbox{Ni}}_{0.98} {\hbox{Mo}}_{0.02}$ as a function of long-range order parameter $\eta$ are shown in Fig. 2. The dashed lines refer to the equilibrium volume of undoped FeNi. The volumes of the *M*-doped FeNi generally decrease slightly as a function of $\eta$, which means that the equilibrium volume of the ordered phase ($\eta = 1$) is always smaller than that of the random phase. Results for the tetragonal lattice parameters of the ordered ${\hbox{L1}}_{0}\,{\hbox{Fe}}_{1-x}{M}_{x}{\text{Ni}}$, ${\hbox{FeNi}}_{1-x}{M}_{x}$, ${\hbox{Fe}}_{0.98} {\hbox{Mo}}_{0.02}{\text{Ni}}$, ${\hbox{FeNi}}_{0.98} {\hbox{Mo}}_{0.02}$, ${\hbox{Fe}}_{0.93} {\hbox{Co}}_{0.05} {\hbox{Mo}}_{0.02}{\text{Ni}}$ and ${\hbox{Fe}}_{0.95} {\hbox{Co}}_{0.05} {\hbox{Ni}}_{0.98} {\hbox{Mo}}_{0.02}$, as well as the volume changes relative to ${\hbox{L1}}_{0}$ FeNi are listed in Table 1.

From Fig. 2 and Table 1, we can see that only two alloy systems have smaller equilibrium volumes than pure FeNi in the ordered phase: ${\hbox{Fe}}_{1-x} {\hbox{Cr}}_{x}{\text{Ni}}$ and ${\hbox{Fe}}_{1-x} {\hbox{Co}}_{x}{\text{Ni}}$. Furthermore, we observe that Fe substitution leads to smaller equilibrium volumes compared to Ni substitution. This is due to the stronger decrease of the total magnetic moment when Fe is replaced by a dopant compared to ${\hbox{FeNi}}_{1-x}{M}_{x}$. More Fe means larger total magnetic moment, and systems with large magnetic moments tend to have large equilibrium volumes due to the magnetic pressure. In addition, we find that Al, Ti, and Mo increase the volume, which is consistent with the large atomic radii of Al, Ti, and Mo as compared to those of Fe and Ni. The volume increase is the largest for the ${\hbox{FeNi}}_{1-x} {\hbox{Ti}}_{x}$ alloys, which is consistent with the above observation. The large volumes of the ${\hbox{FeNi}}_{1-x} {\hbox{Ti}}_{x}$ alloys are accompanied by large *c*/*a* ratios. Compared to the undoped FeNi alloy, Cr increases the volumes when doping the Ni layer and decreases them when doping the Fe layer. This doping induced reduction of the volume happens because the atomic radii of Cr and Co are not as big as those of Al and Ti and the decreasing Fe content lowers the total magnetic moment, which in turn decreases the equilibrium volume. Cobalt addition negligibly affects the volume when doping on the Ni layer. There is practically no volume change in Fig. 2 and also changes in the magnetic moments are quite small as Table 2 shows. According to the present data, Co can substitute Ni almost perfectly, which is attributable to the fact that Ni and Co are similar chemically. However, Co decreases the volume of FeNi when doping the Fe site.

Total and atomic spin magnetic moments of *M*-doped FeNi are shown in Table 2 for the ordered ($\eta = 1$) and fully random ($\eta = 0$) phases. The bottom rows of both tables show the magnetic moments of undoped FeNi for comparison. The total magnetic moments of *M*-doped FeNi are naturally decreased when Fe is replaced by the dopant. Likewise, there is a slight reduction in the magnetic moments when Ni gets replaced, except when doping with Co. All dopants except Co show antiferromagnetic coupling, although for Al, Ti and Mo the moments of the dopants are very small.

It should be noted that doping with Cr causes bigger reductions in total magnetic moment than the non-magnetic Al, Ti, and Mo dopants. Two factors contribute to this. Firstly, the Cr-doped alloys have smaller equilibrium volumes compared to the Al, Ti, and Mo-doped alloys, which means reduced moment due to the magneto-volume effect. Secondly, as Table 2 shows, Cr favors strong antiferromagnetic coupling, which reduces the total magnetic moment.

**Table 2:** The total magnetic moments (*m*) and partial magnetic moments ($m_{\mathrm {Fe}}$, $m_{\mathrm {Ni}}$, $m_{\mathrm {M}}$) (in unit of $\mu _{\mathrm {B}}$ per atom) of ${\hbox{Fe}}_{1-x}{M}_{x}{\text{Ni}}$ and ${\hbox{FeNi}}_{1-x}{M}_{x}$ ($x = 0.05$, 0.10) (${M} = {\hbox{Al}}$, Ti, Cr, Co), and ${\hbox{Fe}}_{0.98} {\hbox{Mo}}_{0.02}{\text{Ni}}$, ${\hbox{FeNi}}_{0.98} {\hbox{Mo}}_{0.02}$, ${\hbox{Fe}}_{0.93} {\hbox{Co}}_{0.05} {\hbox{Mo}}_{0.02}{\text{Ni}}$ and ${\hbox{Fe}}_{0.95} {\hbox{Co}}_{0.05} {\hbox{Ni}}_{0.98} {\hbox{Mo}}_{0.02}$ for ordered ($\eta = 1$) and random ($\eta = 0$) phases.

Structures	$\eta$ = 1					$\eta$ = 0				
	*m*	$m_{\mathrm {Fe}}$	$m_{\mathrm {Ni}}$	$m_{M}$		*m*	$m_{\mathrm {Fe}}$	$m_{\mathrm {Ni}}$	$m_{M}$	
${\hbox{Fe}}_{0.95} {\hbox{Al}}_{0.05}{\hbox{Ni}}$	1.526	1.233	0.296	− 0.002		1.503	1.217	0.288	− 0.002	
${\hbox{Fe}}_{0.90} {\hbox{Al}}_{0.10}{\hbox{Ni}}$	1.436	1.157	0.283	− 0.004		1.404	1.139	0.270	− 0.005	
${\hbox{Fe}}_{0.95} {\hbox{Ti}}_{0.05}{\hbox{Ni}}$	1.494	1.221	0.286	− 0.013		1.471	1.204	0.279	− 0.012	
${\hbox{Fe}}_{0.90} {\hbox{Ti}}_{0.10}{\hbox{Ni}}$	1.375	1.137	0.263	− 0.025		1.344	1.115	0.253	− 0.024	
${\hbox{Fe}}_{0.95} {\hbox{Cr}}_{0.05}{\hbox{Ni}}$	1.462	1.227	0.287	− 0.052		1.427	1.203	0.279	− 0.055	
${\hbox{Fe}}_{0.90} {\hbox{Cr}}_{0.10}{\hbox{Ni}}$	1.322	1.150	0.269	− 0.096		1.262	1.113	0.254	− 0.105	
${\hbox{Fe}}_{0.95} {\hbox{Co}}_{0.05}{\hbox{Ni}}$	1.601	1.248	0.311	0.042		1.577	1.232	0.305	0.040	
${\hbox{Fe}}_{0.90} {\hbox{Co}}_{0.10}{\hbox{Ni}}$	1.582	1.185	0.312	0.084		1.554	1.169	0.305	0.081	
${\hbox{Fe}}_{0.98} {\hbox{Mo}}_{0.02}{\hbox{Ni}}$	1.560	1.271	0.295	− 0.006		1.536	1.252	0.290	− 0.007	
${\hbox{Fe}}_{0.93} {\hbox{Co}}_{0.05} {\hbox{Mo}}_{0.02}{\hbox{Ni}}$	1.542	1.210	0.298	0.041	(Co)	1.513	1.190	0.290	0.039	(Co)
				$-\,0.006$	(Mo)				$-\,0.007$	(Mo)
${\hbox{FeNi}}_{0.95} {\hbox{Al}}_{0.05}$	1.575	1.295	0.283	− 0.003		1.548	1.276	0.275	− 0.002	
${\hbox{FeNi}}_{0.90} {\hbox{Al}}_{0.10}$	1.534	1.280	0.260	− 0.006		1.496	1.255	0.246	− 0.005	
${\hbox{FeNi}}_{0.95} {\hbox{Ti}}_{0.05}$	1.544	1.281	0.278	− 0.014		1.517	1.263	0.267	− 0.013	
${\hbox{FeNi}}_{0.90} {\hbox{Ti}}_{0.10}$	1.472	1.252	0.249	− 0.029		1.435	1.229	0.231	− 0.025	
${\hbox{FeNi}}_{0.95} {\hbox{Cr}}_{0.05}$	1.499	1.279	0.279	− 0.059		1.473	1.262	0.267	− 0.055	
${\hbox{FeNi}}_{0.90} {\hbox{Cr}}_{0.10}$	1.385	1.247	0.254	− 0.116		1.351	1.226	0.231	− 0.106	
${\hbox{FeNi}}_{0.95} {\hbox{Co}}_{0.05}$	1.637	1.308	0.293	0.035		1.626	1.294	0.292	0.040	
${\hbox{FeNi}}_{0.90} {\hbox{Co}}_{0.10}$	1.654	1.305	0.277	0.072		1.645	1.289	0.276	0.080	
${\hbox{FeNi}}_{0.98} {\hbox{Mo}}_{0.02}$	1.578	1.292	0.294	− 0.008		1.555	1.276	0.285	− 0.007	
${\hbox{Fe}}_{0.95} {\hbox{Co}}_{0.05} {\hbox{Ni}}_{0.98} {\hbox{Mo}}_{0.02}$	1.559	1.230	0.296	0.041	(Co)	1.532	1.214	0.285	0.039	(Co)
				$-\,0.008$	(Mo)				$-\,0.007$	(Mo)
FeNi	1.620	1.311	0.309			1.599	1.295	0.304		

**Figure 3:**
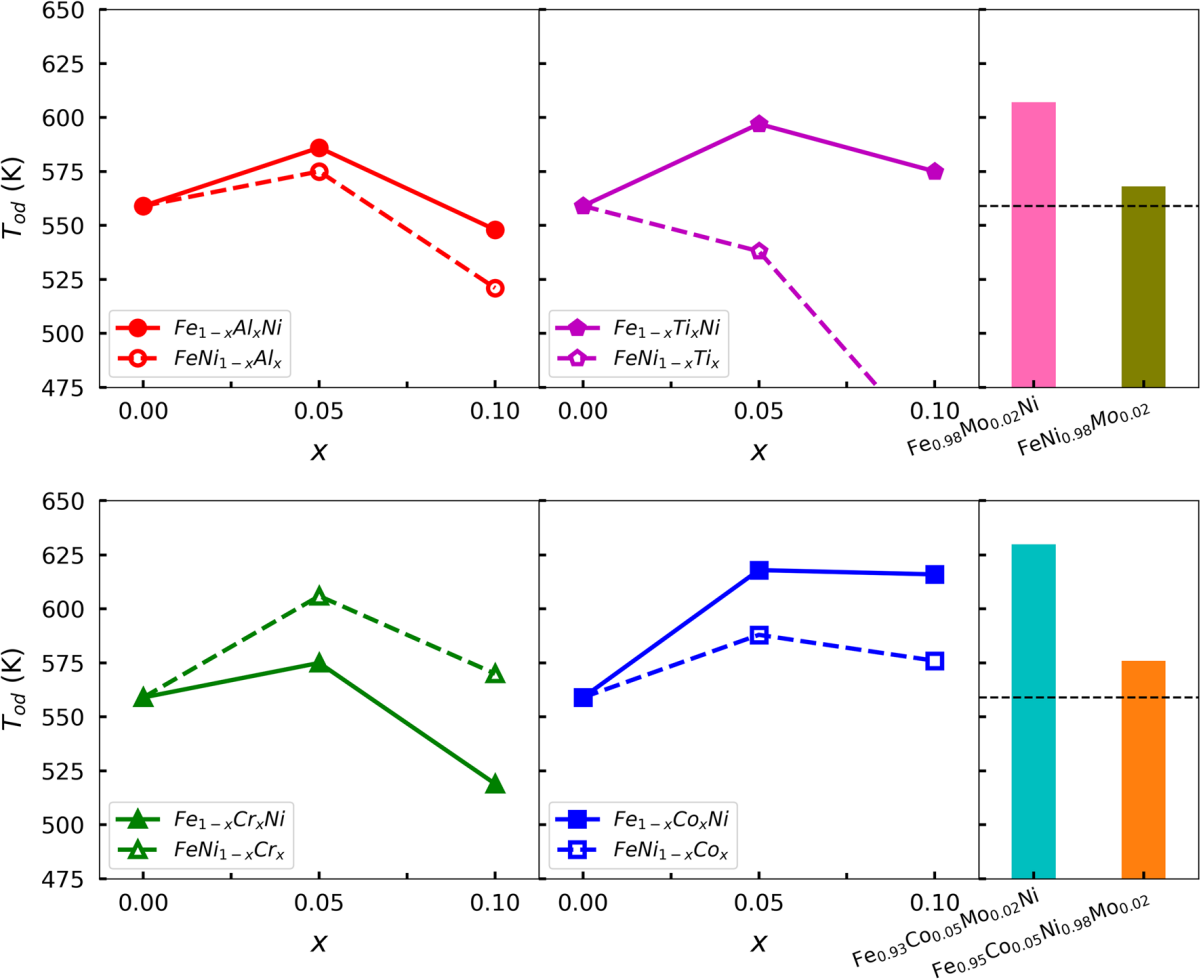
The order–disorder transition temperatures of ${\hbox{Fe}}_{1-x}{M}_{x}{\text{Ni}}$ and ${\hbox{FeNi}}_{1-x}{M}_{x}$ (${M} = {\hbox{Al}}$, Ti, Cr, Co), as well as ${\hbox{Fe}}_{0.98} {\hbox{Mo}}_{0.02}{\text{Ni}}$, ${\hbox{FeNi}}_{0.98} {\hbox{Mo}}_{0.02}$, ${\hbox{Fe}}_{0.93} {\hbox{Co}}_{0.05} {\hbox{Mo}}_{0.02}{\text{Ni}}$ and ${\hbox{Fe}}_{0.95} {\hbox{Co}}_{0.05} {\hbox{Ni}}_{0.98} {\hbox{Mo}}_{0.02}$ as a function of the *M* concentrations. The dashed lines represent the $\hbox{T}_{{od}}$ (559 K) of ${\hbox{L}}1_{0}$ FeNi in the last column.

In our previous paper, we established the order–disorder transition temperature $\hbox{T}_{{od}}$ of undoped FeNi to be 559 K^15^. Here our goal is to understand in what way alloying affects this temperature, and we are specifically looking for ways to increase this transition temperature. The present theoretical order–disorder transition temperatures $\hbox{T}_{{od}}$ of ${\hbox{Fe}}_{1-x}{M}_{x}{\text{Ni}}$ and ${\hbox{FeNi}}_{1-x}{M}_{x}$ alloys, as well as ${\hbox{Fe}}_{0.98} {\hbox{Mo}}_{0.02}{\text{Ni}}$, ${\hbox{FeNi}}_{0.98} {\hbox{Mo}}_{0.02}$, ${\hbox{Fe}}_{0.93} {\hbox{Co}}_{0.05} {\hbox{Mo}}_{0.02}{\text{Ni}}$ and ${\hbox{Fe}}_{0.95} {\hbox{Co}}_{0.05} {\hbox{Ni}}_{0.98} {\hbox{Mo}}_{0.02}$ are given as a function of dopant concentration *M* in Fig. 3. The order–disorder transition temperature $\hbox{T}_{{od}}$ changes differently depending on whether we dope the Fe layer or the Ni layer. A general feature we can identify is that in most cases doping the Fe layer leads to higher transition temperatures compared to doping the Ni layer. Only the Cr case is such where doping the Fe layer decreases the transition temperature compared to Ni layer doping. We ascribe this to the strongly antiferromagnetic nature of Cr.

The changes in the equilibrium volume shows interesting correlation with the transition temperature. For Al, ${\hbox{Fe}}_{1-x} {\hbox{Al}}_{x}{\text{Ni}}$ with smaller volume has a larger transition temperature, while ${\hbox{FeNi}}_{1-x} {\hbox{Al}}_{x}$ with bigger volume has a smaller transition temperature. The ${\hbox{FeNi}}_{1-x} {\hbox{Ti}}_{x}$ alloy, which has very large equilibrium volume at high doping levels, shows rapid decrease of the transition temperature. The ${\hbox{FeNi}}_{1-x} {\hbox{Co}}_{x}$ alloy on the other hand has small equilibrium volumes, but increased transition temperatures.

**Figure 4:**
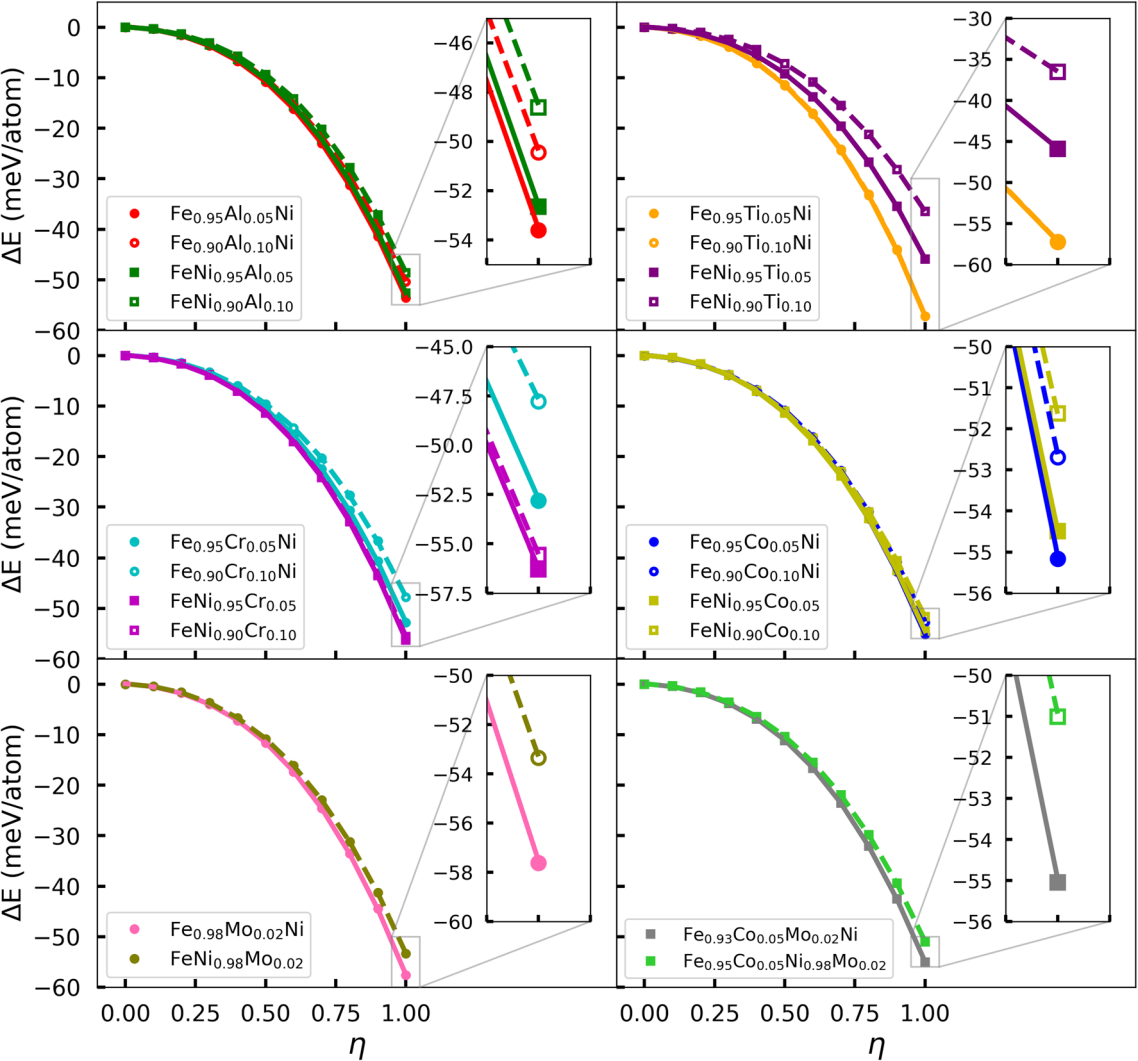
The energies of ${\hbox{Fe}}_{1-x}{M}_{x}{\text{Ni}}$ and ${\hbox{FeNi}}_{1-x}{M}_{x}$ (${M} = {\hbox{Al}}$, Ti, Cr, Co) relative to the energies of fully disordered phases, as well as ${\hbox{Fe}}_{0.98} {\hbox{Mo}}_{0.02}{\text{Ni}}$, ${\hbox{FeNi}}_{0.98} {\hbox{Mo}}_{0.02}$, ${\hbox{Fe}}_{0.93} {\hbox{Co}}_{0.05} {\hbox{Mo}}_{0.02}{\text{Ni}}$ and ${\hbox{Fe}}_{0.95} {\hbox{Co}}_{0.05} {\hbox{Ni}}_{0.98} {\hbox{Mo}}_{0.02}$ as a function of order parameter.

In all alloy cases, except ${\hbox{FeNi}}_{1-x} {\hbox{Ti}}_{x}$, the maximum $\hbox{T}_{{od}}$ occurs around $x=0.05$. We expect the initial increase of $\hbox{T}_{{od}}$ for small values of *x* to be caused by the additional configurational entropy created by the dopant. Due to doping the configurational entropy of the ordered state is larger than zero. For small values of *x*, the proportional entropy gain of the ordered state should be larger than that of the random state. The entropy of the ordered state stabilizes the ordered configuration, as compared to the random state, which leads to the increasing $\hbox{T}_{{od}}$ for small values of *x*. Beyond $x=0.05$, $\hbox{T}_{{od}}$ starts decreasing, because the DFT total energy difference between ordered and random states starts to decrease significantly, as shown in Fig. 4. By inspection of the DFT energy differences one can explain why the $\hbox{T}_{{od}}$ of ${\hbox{Fe}}_{1-x} {\hbox{Ti}}_{x}{\text{Ni}}$, ${\hbox{Fe}}_{1-x} {\hbox{Co}}_{x}{\text{Ni}}$, and ${\hbox{FeNi}}_{1-x} {\hbox{Co}}_{x}$ alloys does not decrease significantly when going from $x=0.05$ to $x=0.10$. Similarly to ${\hbox{Fe}}_{1-x} {\hbox{Co}}_{x}{\text{Ni}}$ alloys, ${\hbox{FeNi}}_{1-x} {\hbox{Cr}}_{x}$ have negligible energy differences. In the ${\hbox{FeNi}}_{1-x} {\hbox{Cr}}_{x}$ case, Fig. 3 shows a sizable $\hbox{T}_{{od}}$ decrease when going from $x=0.05$ to $x=0.10$, but this decrease is smaller than that of ${\hbox{Fe}}_{1-x} {\hbox{Cr}}_{x}{\text{Ni}}$ alloys, which exhibit significant energy differences.

**Figure 5:**
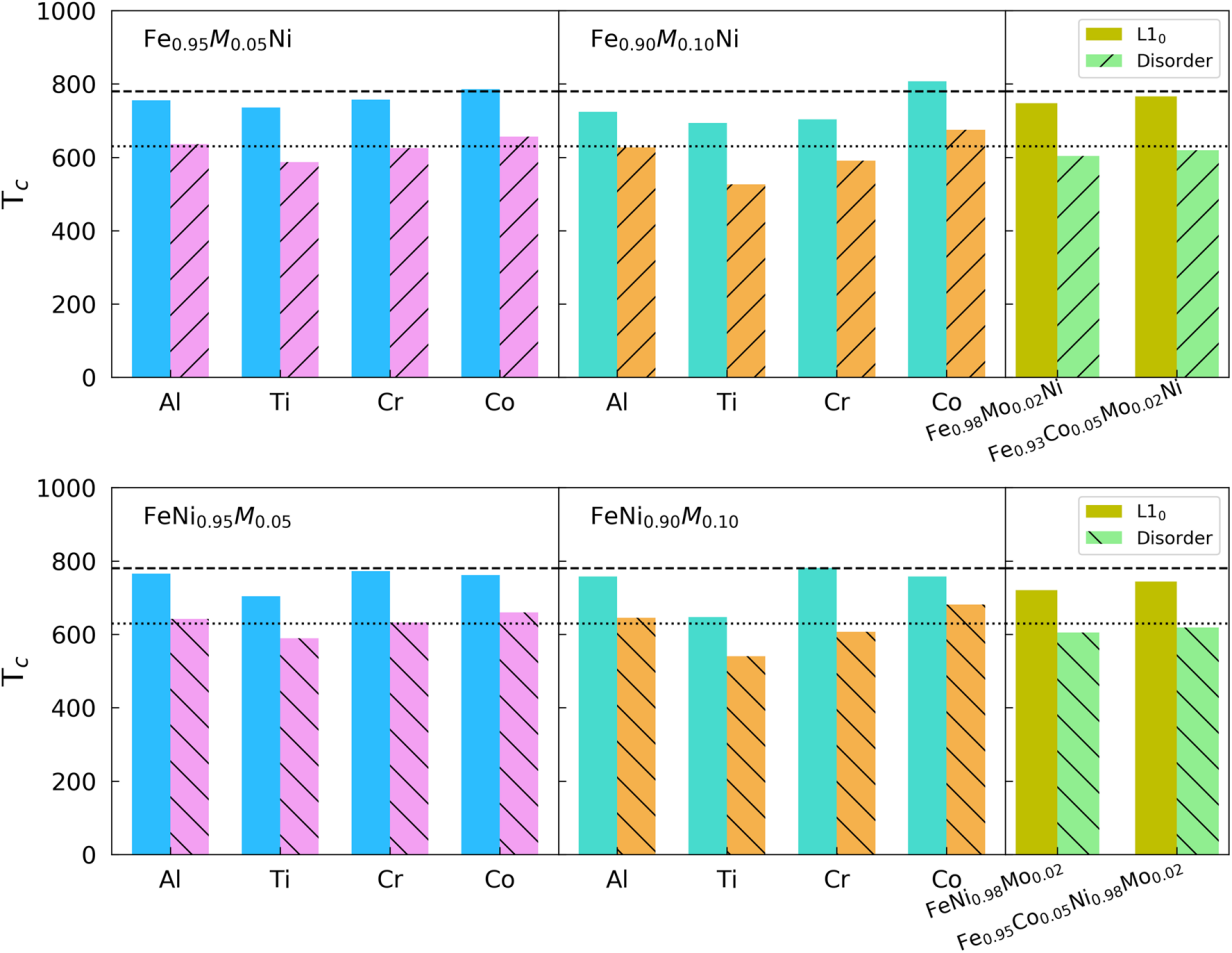
The Curie temperatures of ${\hbox{Fe}}_{1-x}{M}_{x}{\text{Ni}}$ and ${\hbox{FeNi}}_{1-x}{M}_{x}$ (${M} = {\hbox{Al}}$, Ti, Cr, Co), and ${\hbox{Fe}}_{0.98} {\hbox{Mo}}_{0.02}{\text{Ni}}$, ${\hbox{FeNi}}_{0.98} {\hbox{Mo}}_{0.02}$, ${\hbox{Fe}}_{0.93} {\hbox{Co}}_{0.05} {\hbox{Mo}}_{0.02}{\text{Ni}}$ and ${\hbox{Fe}}_{0.95} {\hbox{Co}}_{0.05} {\hbox{Ni}}_{0.98} {\hbox{Mo}}_{0.02}$ are shown. The dashed lines show the $\hbox{T}_c$ (780 K) of ${\hbox{L1}}_{0}$ FeNi, and the dotted lines show the $\hbox{T}_{c}$ (630 K) of fully random FeNi, respectively.

In Fig. 5, the Curie temperatures ${\hbox{T}}_{c}$ are presented for different alloy systems at ordered ($\eta = 1$) and disordered ($\eta = 0$) phases, respectively. All ordered phases have higher $\hbox{T}_c$ than the disordered phases. This predicts that the positive magnetic contribution are likely to increase the order–disorder transition temperature $\hbox{T}_{{od}}$. ${\hbox{Fe}}_{0.90} {\hbox{Co}}_{0.10}{\text{Ni}}$ alloy has the highest Curie temperatures, which is to say that adding Co into the alloy raises the Curie temperature, which is sensible given the rather high ($\approx {1400}$ K) Curie temperature of pure Co. The Curie temperature of ${\hbox{L1}}_{0}\,{\hbox{Fe}}_{0.93} {\hbox{Co}}_{0.05} {\hbox{Mo}}_{0.02}{\text{Ni}}$ is by only 14.2 K smaller than that of ${\hbox{L1}}_{0}$ FeNi.

**Figure 6:**
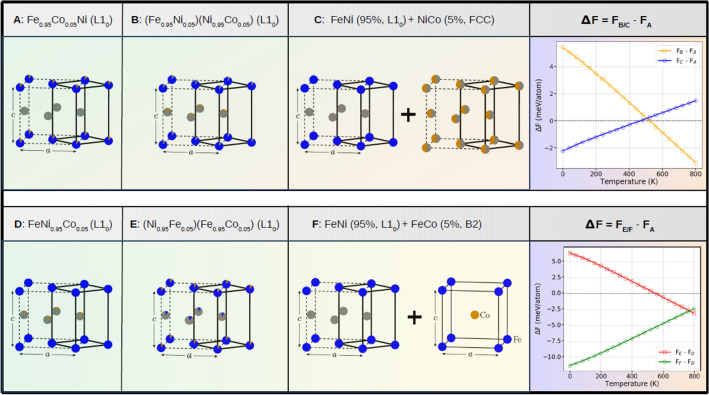
Different types of scenarios which would happen in experiments. Cases *A*, *B* and *C* represent doping on Fe site, and cases *D*, *E* and *F* represent doping on Ni site, respectively. The last column shows the free energy difference between cases *B*/*C* (*E*/*F*) and *A* (*D*).

The highest theoretical order–disorder transition temperatures predicted in the present study are for ${\hbox{Fe}}_{0.95} {\hbox{Co}}_{0.05}{\text{Ni}}$ (618 K) and ${\hbox{Fe}}_{0.93} {\hbox{Co}}_{0.05} {\hbox{Mo}}_{0.02}{\text{Ni}}$ (630 K). In both cases, it is assumed that Co and Mo replace Fe within the Fe sublattice. In practice, however, the situation could be more complex. Alloying additions could induce Fe and Ni intermixing or phase decomposition. In the following, we study the energetics of two chemical processes which might prevent the formation of the ${\hbox{L}}1_{0}$ phase with Co located on the Fe site in ${\hbox{Fe}}_{0.95} {\hbox{Co}}_{0.05}{\text{Ni}}$. In Fig. 6, we consider two different types of scenarios for both Ni-rich and Fe-rich cases. The initial ${\hbox{L}}1_{0}\,{\hbox{Fe}}_{0.95} {\hbox{Co}}_{0.05}{\text{Ni}}$ (case *A*) can change to ${\hbox{Fe}}_{0.95} {\hbox{Ni}}_{0.05}$ on the Fe layer and ${\hbox{Ni}}_{0.95} {\hbox{Co}}_{0.05}$ on the Ni layer (case *B*). Alternatively, the initial ${\hbox{L}}1_{0}\,{\hbox{Fe}}_{0.95} {\hbox{Co}}_{0.05}{\text{Ni}}$ can separate into pure ${\hbox{L}}1_{0}$ FeNi phase plus ferromagnetic fcc NiCo (case *C*). Similarly, the initial ${\hbox{L}}1_{0}\,{\hbox{FeNi}}_{0.95} {\hbox{Co}}_{0.05}$ (case *D*) can change to ${\hbox{Fe}}_{0.95} {\hbox{Co}}_{0.05}$ on the Fe layer and ${\hbox{Ni}}_{0.95} {\hbox{Fe}}_{0.05}$ on the Ni layer (case *E*), or can separate into pure ${\hbox{L}}1_{0}$ FeNi phase plus ferromagnetic B2 FeCo (case *F*). We compute the free energy for each of these processes and check the phase stability as a function of temperature. In this study, the free energies include the configurational and vibrational contributions, but neglect the electronic and magnetic contributions.

First, we discuss the Co doping on the Fe site (cases *A*, *B*, and *C*). According to the free energy differences in Fig. 6, at low temperatures the ordered structure (case *A*) is more stable than the one with interlayer mixing (case *B*), but less stable than the phase separated case (case *C*). At elevated temperatures (above $\sim 500$ K) these trends are reversed so that the ordered single phase structure is more stable than the phase separation, and the interlayer mixing becomes more stable than the ordered structure. Next, we repeat the Co doping for the Ni site (cases *D*, *E*, and *F*). The overall trends are similar to the doping on the Fe site case. Therefore, the thermodynamic stability seems to prevent the formation of the ideal ${\hbox{L}}1_{0}\,{\hbox{Fe}}_{0.95} {\hbox{Co}}_{0.05}{\text{Ni}}$ or ${\hbox{FeNi}}_{0.95} {\hbox{Co}}_{0.05}$ phases. Considering that phase separation requires substantial diffusion, perhaps its impact is less relevant around the ordering temperature. However, the interlayer mixing, which is mainly driven by the configurational entropy, is predicted to affect the Co partition and thus the largest achievable ordering temperature for the Co-doped FeNi system might be somewhat below the largest values from Fig. 3.

## Conclusions

We have studied the effect of alloying on the properties of tetragonal FeNi. Our results show that by alloying it is possible to manipulate the order–disorder transition temperature of FeNi without deteriorating the magnetic properties of the ordered FeNi. We have identified some key insights about how alloying affects the transition temperature. Our results show that the transition temperature increases as a function of dopand concentration up to $x \approx 0.05$. The order–disorder transition temperatures of ${\hbox{Fe}}_{0.95} {\hbox{Co}}_{0.05}{\text{Ni}}$ and ${\hbox{Fe}}_{0.93} {\hbox{Co}}_{0.05} {\hbox{Mo}}_{0.02}{\text{Ni}}$ are 618, 630 K, respectively, which are the highest in the present study. Further alloying causes the transition temperature to decrease. For most dopands studied here substituting Fe leads to a higher transition temperature compared to Ni substitution. At temperatures considered here, Co prefers occupying the Fe site in the Ni-rich ${\hbox{L1}}_{0}\,{\hbox{Fe}}_{0.95} {\hbox{Co}}_{0.05}{\text{Ni}}$ alloy.

## Methods

The first-principles calculations were performed within the exact-muffin-tin orbitals (EMTO) method^16–18^ based on Density Functional Theory^21^. The *s*, *p*, *d*, and *f* orbitals were included in the EMTO basis sets. The single-electron Kohn–Shan equations were solved by the Green’s function technique and the compositional disorder was treated using the coherent-potential approximation (CPA)^19,20^. The total energies were computed via the full charge density technique^22^. The exchange-correlation functional was approximated by the Perdew, Burke, and Ernzerhof (PBE)^23^ generalized gradient approximation. The magnetic transition temperatures were estimated using the UppASD spin dynamics code^24^.

The free energies of ordered, partially ordered and disordered ${\hbox{Fe}}_{1-x}{M}_{x}{\text{Ni}}$ and ${\hbox{FeNi}}_{1-x}{M}_{x}$ phases were expressed as a function of $\eta$,

1 $$\begin{aligned} \begin{aligned} F (V, T, \eta , x) &=E_{0K} (V, \eta , x) - T S_{conf} (\eta , x) + F_{vib} (V, T, \eta , x) + F_{el} (V, T, \eta , x) + F_{mag} (V, T, \eta , x) \end{aligned} \end{aligned}$$

where $E_{0 K}$ is the internal energy per unit cell at 0 K, $S_{conf}$ is the configurational entropy, $F_{vib}$, $F_{el}$ and $F_{mag}$ are the vibrational, electronic and magnetic free energies, respectively. According to the static Concentration Waves method^25^, the configurational entropy of ${\hbox{L1}}_{{0}}\,{\hbox{Fe}}_{1-c}({\hbox{Ni}}_{1-x}{M}_{x})_{c}$ (or (${\hbox{Fe}}_{1-x}{M}_{x})_{1-c} {\hbox{Ni}}_{{c}}$) were described as a function of LRO parameter $\eta$ in the form

2 $$\begin{aligned} \begin{aligned} S_{conf} (\eta , c, x) =&\frac{1}{N} \Big [ 2 \times c \times (1+ \eta ) \times \ln [c \times (1+ \eta )] + 2 \times c \times (1- \eta ) \times \ln [c \times (1- \eta )] \\&+ 2 \times (1-c) \times x \times (1+ \eta ) \times \ln [(1-c) \times x \times (1 + \eta )] \\&+ 2 \times (1-c) \times x \times (1- \eta ) \times \ln [(1-c) \times x \times (1- \eta )] \\&+ 2 \times (1-c) \times (1-x) \times (1 + \eta ) \times \ln [(1-c) \times (1-x) \times (1 + \eta )] \\&+ 2 \times (1-c) \times (1-x) \times (1- \eta ) \times \ln [(1-c) \times (1-x) \times (1- \eta )] \Big ]. \end{aligned} \end{aligned}$$

Here the atomic fraction of the solute *c* equals to 0.5 and *x* is 0.05 or 0.10. Total atomic number *N* equals to 4 in ${\hbox{L1}}_{{0}}$ structure. Detailed information about the approach can be found in Ref.^25^.

The vibrational contribution to Helmholtz free energy, $F_{vib} (V, T, \eta , x)$ = $E_{vib} - TS_{vib}$, was described by Debye model with the Debye temperatures determined by the tetragonal elastic parameters. The electronic contribution to free energy was estimated by $F_{el} \approx - \frac{1}{2} T S_{el} (V, \eta , x) \approx - \frac{2 \pi ^2}{3} k_{B}^{2} T^{2} N_{el} (\varepsilon _{F}, \eta , x)$, where electronic density of state $N_{el} (\varepsilon _{F}, \eta )$ is approximated to be constant in the neighborhood of the Fermi level $\epsilon _{F}$. The magnetic contribution to free energy, $F_{mag} (V, T, \eta , x)$ = $-T S_{mag} (V, T, \eta , x) = - T \frac{\partial \langle H_{mag} (V, T, \eta , x) \rangle }{\partial T}$, and Heisenberg exchange Hamiltonian $H_{mag} (V, T, \eta , x) = - \frac{1}{2} \sum _{i \ne j} J_{ij} \mu _{i} \mu _{j} {\hat{e}}_{i} {\hat{e}}_{j}$ where $J_{ij}$ is the Heisenberg exchange interaction between atoms *i* and *j*, and $\mu _{i}$ and $\mu _{j}$ are the local magnetic moments on sites *i* and *j*. The order–disorder temperature $T_{\mathrm{od}}$ was then obtained by computing $\partial \eta / \partial T$.
